# Impact of Scleral Lenses on Visual Acuity and Ocular Aberrations in Corneal Ectasia: A Comprehensive Review

**DOI:** 10.3390/jpm14101051

**Published:** 2024-10-11

**Authors:** Vincenzo Barone, Daniele Petrini, Sebastiano Nunziata, Pier Luigi Surico, Claudia Scarani, Francesco Offi, Valentina Villani, Marco Coassin, Antonio Di Zazzo

**Affiliations:** 1 Ophthalmology, Campus Bio-Medico University, 00128 Rome, Italy; 2 Ophthalmology Operative Complex Unit, Campus Bio-Medico University Hospital Foundation, 00128 Rome, Italy; 3 Department of Sciences, Optometry and Optics, Roma Tre University, 00144 Rome, Italy; 4 Schepens Eye Research Institute of Massachusetts Eye and Ear, Harvard Medical School, Boston, MA 02114, USA; 5 Rare Corneal Diseases Center, Campus Bio-Medico University Hospital Foundation, 00128 Rome, Italy

**Keywords:** higher order aberrations (HOAs), keratoconus, pellucid marginal degeneration, post-LASIK ectasia, scleral lenses, visual acuity, wavefront-guided scleral lenses

## Abstract

Corneal ectasias, including keratoconus (KC), pellucid marginal degeneration (PMD), and post-LASIK ectasia, poses significant visual rehabilitation challenges due to the resultant irregular astigmatism, myopia, and higher-order aberrations (HOAs). These conditions often resist traditional corrective methods, necessitating advanced optical solutions. Scleral lenses (SLs) have emerged as a primary non-surgical option for managing these complex corneal irregularities. SLs form a smooth optical interface by forming a tear-filled chamber between the lens and the cornea, effectively mitigating HOAs and improving both high-contrast and low-contrast visual acuity (VA). This review evaluates the efficacy of SLs in enhancing VA and reducing aberrations in patients with corneal ectasia. It also explores the technological advancements in SLs, such as profilometry and wavefront-guided systems, which enable more precise and customized lens fittings by accurately mapping the eye’s surface and addressing specific visual aberrations. The current body of evidence demonstrates that custom SLs significantly improve visual outcomes across various ectatic conditions, offering superior performance compared to conventional correction methods. However, challenges such as the complexity of fitting and the need for precise alignment remain. Ongoing innovations in SL technology and customization are likely to further enhance their clinical utility, solidifying their role as an indispensable tool in the management of corneal ectasias.

## 1. Introduction

The cornea, as the foremost refractive element in the eye, plays a pivotal role in maintaining clear vision. Any deformities in its structure, such as those caused by keratoconus (KC), pellucid marginal degeneration (PMD), or post-LASIK ectasia, can disrupt this function [1].

KC is a bilateral and asymmetric corneal ectasia characterized by the progressive thinning and protrusion of the cornea, often affecting the central or inferotemporal regions [2,3]. This condition typically begins in puberty and progresses through the third or fourth decade of life, after which, it generally stabilizes [4]. It affects individuals across all ethnicities and genders, with variability in prevalence and incidence due to differing diagnostic criteria. Estimates of KC incidence range from 5 to 23 per 100,000, with a prevalence as high as 265 per 100,000, likely due to improved detection techniques [5,6]. KC pathogenesis is complex and multifactorial, involving genetic, biochemical, biomechanical, and environmental factors [7]. Genetically, KC is linked to various syndromes and shows a higher prevalence among first-degree relatives, suggesting a genetic component; however, the inheritance pattern appears to be polygenic and multifactorial [8,9,10]. Biochemically, KC involves alterations in corneal protein expression, affecting collagen content and leading to keratocyte apoptosis and necrosis, which compromise corneal integrity [11]. Oxidative stress plays a key role, with imbalances in redox homeostasis resulting in elevated oxidative stress markers and reduced antioxidant levels, contributing to corneal thinning and degeneration [12]. Biomechanical changes, including degeneration of proteoglycans surrounding stromal collagen fibrils and alterations in matrix stiffness, further weaken and disorganize collagen fibers, contributing to the corneal curvature changes typical of KC [13]. Environmental factors such as eye rubbing, allergies, and atopy are strongly associated with KC. Elevated immunoglobulin E levels in affected individuals suggest a role for immune-mediated responses in the disease [14]. Clinically, KC presents with corneal protrusion, thinning, a scissors reflex and Fleischer’s ring, and visible corneal nerves, which are observed in over 50% of cases [15]. While clinical signs and slit-lamp findings are crucial, corneal topography remains the gold standard for diagnosing KC, particularly in early stages where a single parameter may not suffice for diagnosis. Pachymetry and corneal aberration assessments are often combined with topography to assist in early diagnosis and monitoring disease progression [16]. Corneal tomography, which provides three-dimensional imaging of the corneal surfaces, has further enhanced the sensitivity and specificity of ectasia detection, surpassing the capabilities of topography alone [17].

In contrast, PMD is a bilateral peripheral corneal ectasia characterized by a narrow band of thinning, typically in the inferior cornea [18,19]. A zone of unaffected cornea, about 1−2 mm wide, separates the thinned area from the limbus. Unlike KC, PMD is often diagnosed later in life, usually between the second and fifth decades, and progresses more slowly [20,21,22]. Statistical studies on PMD are limited, and the condition is likely underreported, often misdiagnosed as KC due to their overlapping clinical features. Epidemiological estimates suggest PMD is rare, with a prevalence of approximately 20 per 100,000 [21]. Histopathological and electron microscopy studies have identified similarities between PMD and KC, such as abnormally spaced collagen fibers [23]. Conditions like obesity and obstructive sleep apnea have been linked to PMD, potentially through hypoxia-induced inflammation, which may contribute to corneal thinning [24]. Furthermore, floppy eyelid syndrome in obese individuals may exacerbate corneal thinning and ectasia [25]. While atopic conditions and systemic diseases have been observed in patients with PMD, no definitive causal relationships have been established [25]. Histological findings in PMD include an absent or irregular Bowman’s membrane, increased stromal mucopolysaccharides, stromal thinning, and irregular collagen bundles, which are also characteristics of advanced KC [26]. Although slit-lamp examinations can sometimes differentiate PMD from KC, especially in early or advanced cases, corneal topography is required for an accurate diagnosis due to the distinct topographic patterns of the two conditions [18,21].

Corneal ectasia can also occur as a complication following LASIK surgery, where progressive corneal steepening and thinning may develop months or even years after the procedure [27,28]. Post-LASIK ectasia can affect both eyes, even in cases without preoperative signs of KC or PMD, though topographic and tomographic assessments might reveal subtle abnormalities indicative of a pre-existing ectatic disorder [29,30,31]. Recent data indicate that the incidence of post-LASIK ectasia has significantly decreased, now estimated to be as low as 33 per 100,000, due to improved screening methods [32]. Post-LASIK ectasia is often considered an iatrogenic condition resulting from LASIK-induced alterations in corneal biomechanics that lead to thinning and ectasia. This structural disruption compromises the biomechanical stability of the cornea [33]. Research suggests that post-LASIK ectasia could be a progression of an underlying predisposition to corneal dilation, exacerbated by LASIK-induced biomechanical changes [34]. Long-term factors such as intraocular pressure, frequent eye rubbing, and blinking also contribute, with eye rubbing, in particular, heightening corneal damage through inflammatory responses and matrix degradation [28].

All these abnormalities introduce severe irregular astigmatism, profound myopia, and higher-order aberrations (HOAs), all of which markedly degrade both the acuity and quality of vision [20,21,31,35,36]. HOAs, in particular, are notoriously detrimental, impacting high-contrast visual acuity (HCVA) as well as low-contrast visual acuity (LCVA). Furthermore, traditional optical aids often fall short in fully correcting these visual impairments [37,38,39,40,41]. Optical strategies for corneal ectasia vary based on the severity of the disorder [42,43,44]. In its milder forms, corrective glasses may suffice. However, as the severity increases, irregular astigmatism and HOAs necessitate advanced corrective measures such as soft spherical, toric, rigid corneal, or scleral lenses (SLs), piggyback systems, and hybrid lenses. SLs are often the solution of choice when other fittings fail to enhance visual acuity (VA) or comfort [7,45,46]. SLs work by creating a tear-filled reservoir between the cornea and the lens, which smooths the corneal surface and compensates for many HOAs, thereby improving the overall visual quality [47,48]. Furthermore, individuals with irregular corneas experience significant improvements in their quality of life (QoL) after being fitted with SLs, as evidenced by enhanced visual function and higher patient-reported QoL scores [49,50,51]. These benefits are particularly pronounced in patients with advanced corneal ectasia, where SLs play a crucial role in maintaining daily activities, regardless of disease severity [52,53].

This review aims to comprehensively evaluate the benefits of SLs in terms of VA and aberrometric patterns in patients with irregular corneas. By examining these parameters, we can better understand the full extent of the visual improvements that SLs can provide and further substantiate their role as a primary non-surgical treatment for corneal ectasias.

## 2. Scleral Lenses

### 2.1. Classification

SLs are large-diameter lenses crafted from rigid, gas-permeable materials, designed to vault over the entire cornea and rest on the conjunctiva overlying the sclera [54]. Their development dates back to the late 19th century with the introduction of the earliest therapeutic blown scleral shells. Adolf Fick first proposed their use for visual correction in 1888 [55]. The introduction of polymethyl methacrylate (PMMA) later popularized the use of SLs, although its lack of oxygen permeability led to complications such as corneal edema and the development of Sattler’s veil [48]. The recognition of the need for oxygen-permeable materials spurred advancements, resulting in lenses that greatly improved wearer comfort and acceptance, and minimized complication rates [48]. Today, contemporary scleral lenses utilize advanced gas-permeable materials, enabling the effective management of a wide array of conditions, from refractive errors to complex ocular surface diseases [56,57,58,59,60,61]. Traditionally, SLs were categorized based on their diameter relative to the visible horizontal iris diameter. Those up to 6 mm were termed mini-scleral lenses, and beyond 6 mm, they were considered large-scleral lenses, while corneo-scleral lenses touched both the cornea and sclera. Modern classification, however, now uniformly refers to these as SLs if they rest on the conjunctiva without touching the cornea [54].

### 2.2. Design

SLs are designed with three distinct zones, each serving a specific function. The optical zone plays a key role in providing refractive correction tailored to the patient’s visual needs, and can integrate wavefront technology to correct specific aberrations, particularly in cases of highly irregular corneas such as KC, PMD, post-LASIK ectasia, and post-transplant conditions [57,62,63,64,65,66,67]. The transitional zone controls the depth of the fluid reservoir between the lens and the cornea, helping to ensure a comfortable fit. Finally, the landing zone is where the lens rests on the sclera, and this area can be customized using ocular impressions or scleral profilometry to optimize the lens’s fit [57,68,69,70].

Despite initial issues such as corneal vascularization, lens decentration, and fogging, advancements in high Dk gas-permeable materials and precision manufacturing have made SLs a safe and effective option for managing irregular corneas [56,67,71,72,73,74,75,76,77,78,79]. Their ability to maintain alignment and elevation ensures corneal protection, minimizes the risk of scarring, and maximizes wearer comfort [53,57,80].

### 2.3. Fitting

The fitting process for SLs differs notably from that of other contact lenses [81]. Unlike corneal lenses, which rely heavily on corneal topography for fitting, SLs rest on the sclera, making corneal topography less useful [80,82]. Currently, the most widely used fitting method involves a trial set of lenses, where various parameters such as overall diameter, sagittal depth, radius, and power are tested to find the most suitable lens [83]. Once the basic fit is established, further adjustments can be made to refine the fit, including modifications to the limbal and peripheral curves, as well as the landing zone. Additionally, visual performance can be optimized by incorporating features such as an aspheric, toric, or wavefront-guided front surface design [57,63,80]. For patients with highly irregular corneal or scleral shapes, the impression technique offers a particularly effective solution [84]. This method involves creating a mold of the ocular surface, allowing for a lens that conforms precisely to both the cornea and sclera [85]. Modern approaches, including corneal profilometry, have further streamlined the process by digitizing the mold and producing highly customized lenses [57,80]. Corneoscleral profilometry allows for a detailed topographic assessment of the cornea, limbus, and anterior sclera, providing valuable quantitative data such as sagittal height, conjunctival irregularities, and scleral and corneal asymmetry. These metrics are essential for selecting and designing a scleral lens tailored to the unique anatomical features of the patient’s eye [57,80,86,87]. These technological advancements have greatly improved the precision and effectiveness of SL fitting, making them a valuable tool in managing complex corneal conditions.

The fitting process also plays a critical role in influencing patient outcomes, particularly in terms of long-term comfort. Recent research has demonstrated the practicality of using corneoscleral profilometry for custom SL fitting. The evidence suggests that profilometry-guided fittings require a similar number of lenses and follow-up visits compared to traditional diagnostic methods, underscoring its efficiency [57]. Further emphasizing the significance of a precise fitting process, a study on scleral asymmetry demonstrated that SLs often do not sit uniformly on the ocular surface. This misalignment can lead to issues such as scleral blanching and discomfort, caused by excessive pressure from the lens. Proper alignment and customized fitting are therefore essential in minimizing these complications and ensuring optimal lens performance and patient comfort. Tailoring the fit to each patient’s unique ocular structure is critical for achieving better clinical outcomes [88].

## 3. Clinical Applications of Scleral Lenses

The use of SLs has been extensively validated as an effective approach to improving VA in patients with irregular corneal conditions. SLs are particularly beneficial for individuals with compromised corneal architecture, offering significant visual rehabilitation. One of the key aspects of SLs is their ability to address HOAs, which are often responsible for debilitating visual distortions that conventional corrective methods, such as spectacles or soft contact lenses, fail to fully correct (Table 1).

### 3.1. Custom Scleral Lenses

The study conducted by Kumar et al. explored the effects of SLs on VA, refraction, and HOAs in patients suffering from post-LASIK ectasia, KC, and PMD. Their findings demonstrated marked improvements in visual outcomes across all patient groups. Corrected distance visual acuity (CDVA) showed a significant improvement, with median values improving from 0.30 logMAR before SL wear to 0.00 logMAR during SL wear (*p* < 0.001). Furthermore, the study revealed notable improvements in refractive outcomes, with the median spherical equivalent decreasing from −2.75 D to −0.50 D and cylindrical refraction dropping from 3.75 D to 0.50 D (both *p* < 0.001). Another critical finding from Kumar et al. was the substantial reduction in HOAs with SL wear. The higher-order root mean square (HO-RMS) error was significantly reduced from 0.90 μm to 0.32 μm (*p* < 0.001), reflecting a dramatic improvement in optical quality. The most pronounced decreases were observed in coma and trefoil aberrations, particularly in the KC group, where these aberrations initially presented at higher levels. These results highlight the capacity of SLs to correct complex optical aberrations, offering enhanced visual performance for patients with irregular corneas [46].

Similarly, Macedo-de-Araújo et al. conducted a comprehensive study evaluating the optical and visual quality outcomes of SLs in patients with both irregular corneas and regular corneas over a 12-month period. The findings strongly supported the efficacy of SLs in improving VA across a range of corneal conditions. In patients with irregular corneas, HCVA improved significantly with SLs, from +0.35 logMAR (habitual correction) and +0.29 logMAR (best spectacle correction) to +0.08 logMAR (*p* < 0.001). A similar trend was observed in patients with regular corneas, with VA improving from +0.17 logMAR (habitual correction) and +0.12 logMAR (best spectacle correction) to +0.10 logMAR with SLs (*p* < 0.05). These results underscore the superior performance of SLs compared to other corrective methods, demonstrating their ability to provide improved visual outcomes for both irregular and regular corneas. An additional benefit of SLs highlighted by this study was the significant reduction in night vision disturbances, as measured by the Light Disturbance Index (LDI). In the irregular cornea group, the LDI dropped from 13.85% (habitual correction) and 15.89% (best spectacle correction) to 5.75% with SLs (*p* < 0.001). In the regular cornea group, the reduction was also notable, with the LDI decreasing from 6.16% (habitual correction) and 5.98% (best spectacle correction) to 3.99% with SLs (*p* < 0.05). These reductions suggest that SLs are particularly effective in enhancing visual comfort in low-light conditions, which is a common challenge for patients with both regular and irregular corneas. The whole eye aberrometry findings were consistent with these improvements, showing significant reductions in HOAs with SL wear. Patients with irregular corneas experienced a 55% reduction in total HOAs, while those with regular corneas saw a less pronounced but still significant reduction. SLs were particularly effective in addressing vertical coma, coma, and secondary astigmatism, contributing to a notable enhancement in overall visual quality. These findings reinforce the idea that SLs offer a comprehensive solution not only for improving VA but also for addressing complex HOAs that are often resistant to traditional correction methods. Subjective assessments further corroborated these objective improvements. Both groups of patients reported significant reductions in visual symptoms such as glare, halos, and starbursts, as assessed by the Quality of Vision (QoV) questionnaire. These subjective improvements remained stable throughout the 12-month follow-up period, indicating the long-term efficacy of SLs in managing visual symptoms and enhancing patient satisfaction. The sustained improvement in visual function over time highlights the potential of SLs as a reliable and durable option for patients with both irregular and regular corneal conditions [89].

Formisano et al. also evaluated the visual benefits of SLs in patients with KC. Their findings demonstrated a significant improvement in VA with SLs, surpassing the performance of both spectacles and rigid gas permeable (RGP) lenses. Specifically, mean logMAR visual acuity improved from 0.2 with glasses and 0.1 with RGP lenses to −0.002 with SLs (*p* < 0.05). This improvement highlights the superior ability of SLs to correct visual impairments in patients with KC, particularly in more advanced cases. Moreover, the patient-reported outcomes indicated high levels of comfort during SL wear, with an average daily wear time exceeding six hours. These results emphasize the practical advantages of SLs, which offer not only enhanced VA but also improved comfort and extended wearability, making them a preferred choice for KC patients [91].

Dutta et al. also examined the impact of SLs on HOAs, CDVA, and contrast sensitivity (CS) in patients with PMD. Their findings revealed a significant improvement in CDVA, with the mean logMAR improving from 0.42 to 0.05 (*p* < 0.001) after SL fitting. This substantial enhancement in VA highlights the efficacy of SLs in addressing the irregular astigmatism and refractive errors typically associated with PMD. Beyond VA, contrast sensitivity also showed marked improvement, increasing from 1.24 to 1.58 (*p* < 0.001). The ability of SLs to enhance contrast sensitivity, a critical component for performing routine activities, especially in low-contrast environments, underscores the comprehensive benefits of these lenses for patients with complex corneal irregularities. In addition, they reported significant reductions in HOAs with SL wear. The HO-RMS error decreased from 0.89 μm to 0.38 μm (*p* < 0.001), indicating a substantial improvement in overall optical quality. Specific aberrations, such as coma and trefoil, exhibited dramatic reductions. Coma improved from 0.45 μm to 0.20 μm (*p* < 0.001), while trefoil decreased from 0.64 μm to 0.13 μm (*p* < 0.001). However, it is worth noting that RMS spherical aberrations did not show a statistically significant improvement, indicating that certain types of HOAs may be less responsive to SL correction. This points to the need for further refinement in SL design to fully address the range of aberrations present in patients with complex corneal conditions [93].

Marty et al. focused their investigation on the efficacy of SLs in managing irregular corneas following refractive surgery, another challenging scenario for visual rehabilitation. Their results indicated significant improvements in VA and ocular surface conditions with the use of SLs. The mean BCVA improved from 0.33 to 0.08 logMAR (*p* < 0.001), demonstrating that SLs are highly effective in restoring vision in patients with post-surgical corneal irregularities. Furthermore, the Ocular Surface Disease Index (OSDI) score, which measure ocular discomfort and symptoms of dry eye, significantly decreased from 66.2 to 42.4 (*p* < 0.001), underscoring the ability of SLs to enhance both visual function and patient comfort. This improvement is especially valuable for individuals suffering from chronic dry eye following refractive surgeries, a common complication that can severely impact QoL. SLs offer a key advantage in managing these symptoms by forming a tear-filled reservoir over the cornea. This reservoir not only enhances VA but also provides continuous hydration to the ocular surface. By stabilizing the tear film and minimizing exposure-related damage, SLs provide a dual solution that addresses both optical correction and patient comfort, ultimately improving overall QoL [58]. In terms of optical quality, Marty et al. reported significant reductions in ocular aberrations with SL wear. The mean ocular scatter index (OSI), which quantifies light scatter in the eye, decreased from 7.2 to 3.0 (*p* < 0.001), indicating a substantial improvement in visual clarity. Additionally, total ocular aberrations were reduced from 2.58 to 1.98 μm (*p* = 0.035), while HOAs decreased from 0.94 to 0.48 μm (*p* = 0.0018). These results further validate the effectiveness of SLs in addressing complex optical disturbances that are often left uncorrected by other modalities. The marked improvement in optical quality, combined with the reduction in visual symptoms such as glare and halos, highlights the comprehensive rehabilitative potential of SLs for patients with post-surgical corneal irregularities [92].

The collective evidence supports that SLs are not merely corrective devices but play a critical role in the rehabilitation of visual function in patients with significant corneal pathologies. The customization of SLs allows for a tailored approach, optimizing outcomes by addressing individual visual deficits. Nonetheless, challenges persist in completely correcting residual HOAs in more complex cases, indicating a need for ongoing innovation and research in SL technology.

### 3.2. Wavefront-Guided Scleral Lenses

Nguyen et al. presented a pivotal case study highlighting the limitations of conventional SLs in managing residual HOAs in patients with severely aberrated eyes, particularly those with KC. The case focused on a “20/20 unhappy” patient—a term used for individuals who, despite achieving seemingly optimal VA, still experience visual disturbances such as halos, glare, and smearing. Conventional SLs are known to mask between 60 and 65% of HOAs; however, the remaining 35–40% of uncorrected HOAs can still lead to visual dissatisfaction, even when the clinical outcome appears satisfactory. In this case, a 40-year-old male with bilateral KC, severe in the right eye and moderate in the left, reported significant dissatisfaction with his overall visual quality, despite achieving a Snellen visual acuity of 20/20 + 2 in the right eye and 20/16 + 2 in the left with conventional SLs. He reported symptoms including nighttime halos, glare, and smearing, with specific complaints of a “Ferris wheel” shadow in his right eye and a “U-shaped” shadow in his left, leading to overall dissatisfaction with the visual performance of conventional SLs. Upon further examination, residual HOAs were identified as the primary cause of the patient’s dissatisfaction, with wavefront measurements revealing higher-than-normal wavefront errors and poor visual image quality metrics. To address these residual aberrations, the patient was fitted with wavefront-guided scleral lenses (wfgSLs). The result was a significant reduction in HO-RMS wavefront error, from 0.49 μm to 0.19 μm in the right eye and from 0.39 μm to 0.25 μm in the left. This reduction in aberrations led to a marked improvement in VA, with the patient achieving 20/10 in the right eye and 20/13 in the left. Additionally, his visual Strehl ratios improved from 0.067 to 0.150 in the right eye and from 0.092 to 0.121 in the left. The patient also experienced significant relief from visual disturbances such as smearing and shadowing [66]. This case underscores the potential benefits of wfgSLs in managing complex cases of KC where conventional SLs may fall short. While standard SLs are highly effective in addressing refractive errors and basic aberrations, they may not be sufficient for patients with severe corneal irregularities who experience residual HOAs.

Marsack et al. provided further insights into the performance of wfgSLs in KC eyes. Specifically, the lower-order RMS error was reduced by 25% (*p* < 0.001) and the higher-order RMS error was reduced by 17% (*p* < 0.02) when compared to conventional spherical equivalent SLs. Notably, while wfgSLs effectively reduced lower-order RMS to levels below those observed in a well-corrected normal population (*p* < 0.001), they were less effective in fully normalizing the higher-order RMS error, with residual HOAs remaining slightly elevated compared to normal levels (*p* = 0.41). In addition to these objective improvements, the study also reported enhancements in visual image quality metrics such as logVSX, logNS, and logLIB, with improvements ranging from 20% to 30%, bringing these values within normal ranges for the majority of participants. However, HCVA did not reach the levels typically seen in normal age-matched populations [63].

Sabesan et al. further explored the feasibility of correcting HOAs in KC using wfgSLs. The study utilized a custom-built Shack–Hartmann wavefront sensor to measure aberrations through a dilated pupil while patients wore conventional SLs. These wavefront measurements were then used to design wfgSLs, accounting for decentration and rotation relative to the pupil. This high level of customization allowed for the precise correction of individual aberration profiles. HOAs were reduced by a factor of 3.1, with the RMS errors decreasing from 1.17 μm to 0.37 μm for a 6 mm pupil. This substantial reduction in HOAs translated into a significant improvement in VA, with an average gain of 1.9 lines. Additionally, contrast sensitivity improved by factors of 2.4, 1.8, and 1.4 at spatial frequencies of 4, 8, and 12 cycles per degree, respectively. Despite these impressive gains, the VA still fell short of normal levels in KC patients, likely due to long-term neural adaptations to distorted retinal images. This suggests that even with optimal optical correction, residual visual limitations may persist in patients with longstanding KC [64].

Hastings et al. also evaluated the performance of wfgSLs in patients with corneal ectasia, including KC and PMD. Their findings mirrored those of Sabesan et al., showing significant reductions in the HO-RMS wavefront error by 43% compared to conventional SLs, and by 71% compared to habitual correction. These reductions led to notable improvements in visual acuity, from +0.12 logMAR with habitual correction to −0.09 logMAR with wfgSLs. Contrast sensitivity also showed a significant enhancement, with the area under the curve increasing by 26% when switching from habitual to conventional lenses, and by another 14% when switching to wfgSLs. The study also highlighted that the improvement in HOAs with wfgSLs was closely correlated with the posterior corneal radius of curvature, indicating that patients with more severe ectasia may benefit most from wavefront-guided corrections [65].

Finally, Rijal et al. explored the potential impact of misalignment in wfgSLs. They simulated misalignment by applying wavefront-guided corrections at two distinct locations: the average decentered location (ADL) and the geometric center (GC) of the lens. The findings revealed a significant decline in visual quality when the correction was misaligned, as demonstrated by an increase in the HO-RMS wavefront error and a reduction in the visual Strehl ratio (logVSX). Notably, the HO-RMS error increased more dramatically at the GC, with an average change of 0.38 μm, compared to a smaller increase of 0.09 μm at the ADL. This rise in aberrations was closely linked to a corresponding drop in visual quality, as the logVSX values were notably worse at the GC than at the ADL in 33 of the 36 eyes evaluated. Additionally, the study assessed the predicted changes in VA resulting from these misalignments. It was observed that misalignment at the geometric center led to a more substantial predicted loss in VA, with 35 out of 36 eyes losing more than three letters on a standard eye chart. In contrast, 19 out of 36 eyes experienced a similar loss when the correction was applied at the ADL. These findings underscore the critical importance of precise, individualized fitting for wfgSLs [90].

These studies collectively emphasize the transformative potential of wfgSLs while also highlighting the technical precision required for optimal outcomes. The ability to customize corrections based on individual aberration profiles provides significant improvements in visual quality, particularly in patients with severe corneal irregularities. However, achieving these benefits relies on accurate alignment and tailoring of the lenses, underscoring the need for advanced fitting techniques and ongoing innovation in SL technology.

## 4. Limitations and Future Directions

SLs have become invaluable in the rehabilitation of visual function for patients with significant corneal pathologies, yet several challenges remain. One of the primary issues is the incomplete correction of residual HOAs, particularly in complex cases such as advanced KC or post-surgical ectasia [90,94,95]. Additionally, patient adaptation and comfort with SLs can vary. While generally well-tolerated, some patients experience issues such as lens decentration, fogging, or discomfort from pressure on the ocular surface, especially in cases with highly irregular corneal topographies [96,97,98]. Furthermore, the fitting of SLs is a complex and costly process that can limit accessibility, particularly in resource-limited settings. The need for specialized equipment and the expertise required for fitting and adjustments can make SLs less accessible. Moreover, the ongoing maintenance and care, such as regular follow-ups and potential lens replacements, add to the burden on both patients and practitioners [99]. However, recent technological innovations, including the development of portable, user-friendly profilometry devices and standardized fitting protocols, offer promise in reducing both the cost and complexity of SL fittings. These advancements could make SLs more accessible to a wider population. Additionally, the increasing use of telemedicine and remote consultations may further extend access by enabling specialists to assist with fittings and adjustments from a distance, potentially expanding the reach of SL technology into underserved areas. Despite these challenges, recent advancements in SL technology, including profilometry and wave-front customization techniques, have greatly improved the precision and effectiveness of these lenses. These innovations enable more tailored designs, improving both comfort and visual outcomes by better matching individual corneal topographies [50,51,52,100]. A patient-centered approach is vital for assessing the long-term success of SLs. Emphasizing patient compliance, particularly in the consistent use and care of their lenses, is critical for achieving optimal therapeutic outcomes. Additionally, collecting long-term follow-up data is essential for evaluating the sustained effectiveness of SLs, especially in chronic conditions. Continuous monitoring and patient feedback offer valuable insights into potential complications, adherence issues, and the overall impact of SLs on QoL.

## 5. Conclusions

In conclusion, this comprehensive review of the existing literature provides compelling evidence that custom SLs are highly effective in enhancing VA and reducing HOAs in patients with irregular corneas, across a wide range of clinical conditions. The consistent findings across the literature demonstrate substantial improvements in both HCVA and LCVA, as well as significant reductions in specific aberration patterns, including coma and trefoil. These results not only underscore the capacity of SLs to markedly improve the quantitative aspects of visual performance, but they also highlight their ability to enhance the qualitative experience of vision, significantly improving patient satisfaction and QoL, thus affirming their critical role in treating corneal ectasias.

## Figures and Tables

**Table 1:** Summary of key studies on SLs and their impact on VA and HOAs.

Year	Research Group	Key Findings
2013	Sabesan et al. [64]	Custom wfgSLs reduced HOAs and improved contrast sensitivity in KC patients, though VA did not reach normal levels.
2014	Marsack et al. [63]	wfgSLs significantly reduced the lower-order and higher-order RMS errors, with visual image quality metrics falling within normal ranges for most participants.
2019	Hastings et al. [65]	wfgSLs showed significant reductions in the HO-RMS wavefront error and notable improvements in VA and contrast sensitivity, particularly in patients with more severe ectasia.
2020	Macedo-de-Araújo et al. [89]	SLs improved VA in both irregular and regular corneas, with significant reductions in night vision disturbances and HOAs. Patients reported decreased visual symptoms such as glare, halos, and starbursts, with improvements maintained over 12 months.
2020	Rijal et al. [90]	Misalignment of wfgSLs significantly degraded visual quality, highlighting the critical importance of accurate alignment for optimal outcomes in wavefront-guided corrections.
2021	Formisano et al. [91]	SLs provided superior VA compared to spectacles and RGP lenses in KC patients, with high levels of comfort and extended wear time.
2021	Kumar et al. [46]	SLs significantly improved CDVA, refractive outcomes, and HOAs in patients with post-LASIK ectasia, KC, and PMD.
2021	Nguyen et al. [66]	wfgSLs reduced the HO-RMS wavefront error and improved VA and visual Strehl ratios in KC patients who were dissatisfied with conventional SLs due to residual HOAs.
2022	Marty et al. [92]	SLs were highly effective in managing irregular corneas post-refractive surgery, improving BCVA, reducing OSDI scores, and decreasing ocular aberrations.
2024	Dutta et al. [93]	SLs significantly improved CDVA, contrast sensitivity, and HOAs in PMD patients.

BCVA: Best Corrected Visual Acuity; CDVA: corrected distance visual acuity; HCVA: high-contrast visual acuity; HO-RMS: higher-order root mean square (error); HOAs: higher-order aberrations; KC: keratoconus; OSDI: Ocular Surface Disease Index; PMD: pellucid marginal degeneration; RGP: rigid gas permeable (lenses); RMS: root mean square; SLs: scleral lenses; VA: visual acuity; wfgSLs: wavefront-guided scleral lenses.

## Data Availability

Not applicable.
